# Supporting teams with designing for dissemination and sustainability: the design, development, and usability of a digital interactive platform

**DOI:** 10.1186/s13012-024-01410-7

**Published:** 2024-12-31

**Authors:** Maura M. Kepper, Allison J. L’Hotta, Thembekile Shato, Bethany M. Kwan, Russell E. Glasgow, Douglas Luke, Andrea K. Graham, Ana A. Baumann, Ross C. Brownson, Brad Morse

**Affiliations:** 1 Prevention Research Center, Brown School, Washington University in St. Louis, 1 Brookings Dr, St. Louis, MO 63130 USA; 2 Department of Physical Medicine and Rehabilitation, University of Colorado School of Medicine, 12631 East 17th Ave., Aurora, CO 80045 USA; 3 Department of Surgery (Division of Public Health Sciences), Washington University School of Medicine, 660 S. Euclid, St. Louis, MO 63110 USA; 4 Department of Emergency Medicine, Colorado Clinical & Translational Sciences Institute, and the Adult & Child Center for Outcomes Research & Delivery Science, University of Colorado School of Medicine, 1890 N. Revere Ct., Aurora, CO 80045 USA; 5 Department of Family Medicine and the Adult & Child Center for Outcomes Research & Delivery Science, University of Colorado School of Medicine, 1890 N. Revere Ct. , Aurora, CO 80045 USA; 6 Center for Public Health Systems Science, Brown School, Washington University in St. Louis, 1 Brookings Dr, St. Louis, MO 63130 USA; 7 Department of Medical Social Sciences, Northwestern University Feinberg School of Medicine, 750 N. Lakeshore Drive, Chicago, IL 60611 USA; 8 Division of General Internal Medicine, Colorado Clinical & Translational Sciences Institute, and the Adult & Child Center for Outcomes Research & Delivery Science, University of Colorado School of Medicine, 1890 N. Revere Ct., Aurora, CO 80045 USA

**Keywords:** Designing for Dissemination, Sustainability, Co-Design, User-Centered Design, Capacity Building, Equity

## Abstract

**Background:**

Designing for Dissemination and Sustainability (D4DS) principles and methods can support the development of research products (interventions, tools, findings) that match well with the needs and context of the intended audience and setting. D4DS principles and methods are not well-known or used during clinical and public health research; research teams would benefit from applying D4DS. This paper presents the development of a new digital platform for research teams to learn and apply a D4DS process to their work.

**Methods:**

A user-centered design (UCD) approach engaged users (*n* = 14) and an expert panel (*n* = 6) in an iterative design process from discovery to prototyping and testing. We led five design sessions using Zoom and Figma software over a 5-month period. Users (71% academics; 29% practitioners) participated in at least 2 sessions. Following design sessions, feedback from users was summarized and discussed to generate design decisions. A prototype was then built and heuristically tested with 11 users who were asked to complete multiple tasks within the platform while verbalizing their decision-making using the ‘think aloud’ procedure. The System Usability Scale (SUS) was administered at the end of each testing session. After refinements to the platform were made, usability was reassessed with 7 of 11 same users to examine changes.

**Results:**

The interactive digital platform (the D4DS Planner) has two main components: 1) the Education Hub (e.g., searchable platform with literature, videos, websites) and 2) the Action Planner. The Action Planner includes 7 interactive steps that walk users through a set of activities to generate a downloadable D4DS action plan for their project. Participants reported that the prototype tool was moderately usable (SUS = 66) but improved following refinements (SUS = 71).

**Conclusions:**

This is a first of its kind tool that supports research teams in learning about and explicitly applying D4DS to their work. The use of this publicly available tool may increase the adoption, impact, and sustainment of a wide range of research products. The use of UCD yielded a tool that is easy to use. This tool's future use and impact will be evaluated with a broader sample of community partners and projects and the tool will continue to be refined and improved.

**Supplementary Information:**

The online version contains supplementary material available at 10.1186/s13012-024-01410-7.

Contributions to the literature

- The use of Designing for Dissemination and Sustainability (D4DS) principles and methods aim to help match the development of research products (interventions, tools, findings) with the needs and context of the intended audience and setting.
- This paper presents a new digital, interactive platform, the D4DS Planner, that was developed using user-centered design processes and was considered easy to use and useful by participants.
- The D4DS Planner provides users with guidance on applying the Fit to Context process framework for D4DS that can increase the use and potential impact of D4DS methods and principles.

## Background

Dissemination and sustainability are two primary pillars of dissemination and implementation (D&I) science. These concepts focus on sharing and maintaining the use of effective interventions over time to enhance equitable impact on health at the population level. Active and intentional dissemination efforts help spread interventions to the right people (i.e., the intended audience) in a way that best meets their needs and preferences [1]. The impact of a research initiative is enhanced through active dissemination – spreading the products of research (e.g., interventions) to potential adopters and influencers using planned strategies and appropriate channels. A common goal of researchers is for effective interventions to be used widely (i.e., scaled-up) in routine practice. To maximize impact, researchers also seek to sustain their intervention, which is defined as the ability to maintain the intervention and its benefits over time [2–4]. While dissemination and sustainment are critical to maximize the impact of research on health and well-being [5], these initiatives can require significant resources and specialized expertise. Funding organizations are beginning to highlight the value of research initiatives focusing on dissemination and sustainability by requiring dissemination plans in grant applications and publishing calls for research focused on sustainability. Our goal was to develop a digital tool, the D4DS Planner, to help clinical and public health researchers and research teams with planning for dissemination and sustainability. The tool aimed to help users learn about and apply Designing for Dissemination and Sustainability (D4DS) principles and methods [5, 6] and operationalize the objectives of the recently introduced Fit to Context (F2C) framework for D4DS [5].

D4DS incorporates principles (guiding beliefs based on an approach) from multiple disciplines (e.g., diffusion of innovations, implementation science, communication, business & marketing, systems science, user-centered design) [5–8]. Using transdisciplinary methods (ways of enacting principles from multiple and diverse disciplines) to engage key partners from the start in product development, as well as early and active dissemination and sustainability planning, is not commonly done in a systematic, consistent way. According to Kwan et al., [5] D4DS considers three key components of what is being designed: the research product, dissemination plan to support adoption, and sustainability plan to facilitate use over time. A research ‘product’ is the innovation a research team is trying to disseminate and sustain, which may include an (evidence-based) intervention, evidence, treatment, technology, model of care, policy, guideline, or implementation strategy [5]. Three central principles of D4DS, include: 1) beginning with the end in mind when planning research initiatives (i.e., who will adopt, implement, sustain, and potentially benefit from the products of research); 2) ensuring innovation-context fit (i.e., that the products of research can be implemented and equitably effective in real-world settings); and 3) planning for active dissemination and sustainability [5, 9]. When a D4DS approach is not used, a research initiative may suffer from poor innovation-context fit, making it less likely that a research product will be adopted or sustained over time, lessening the impact of the research. A mismatch occurs when a research product – or its dissemination and sustainability plans – are not aligned with the priorities, resources, or capacity of the organization, setting, or target audience [10, 11].

**Fig. 1:**
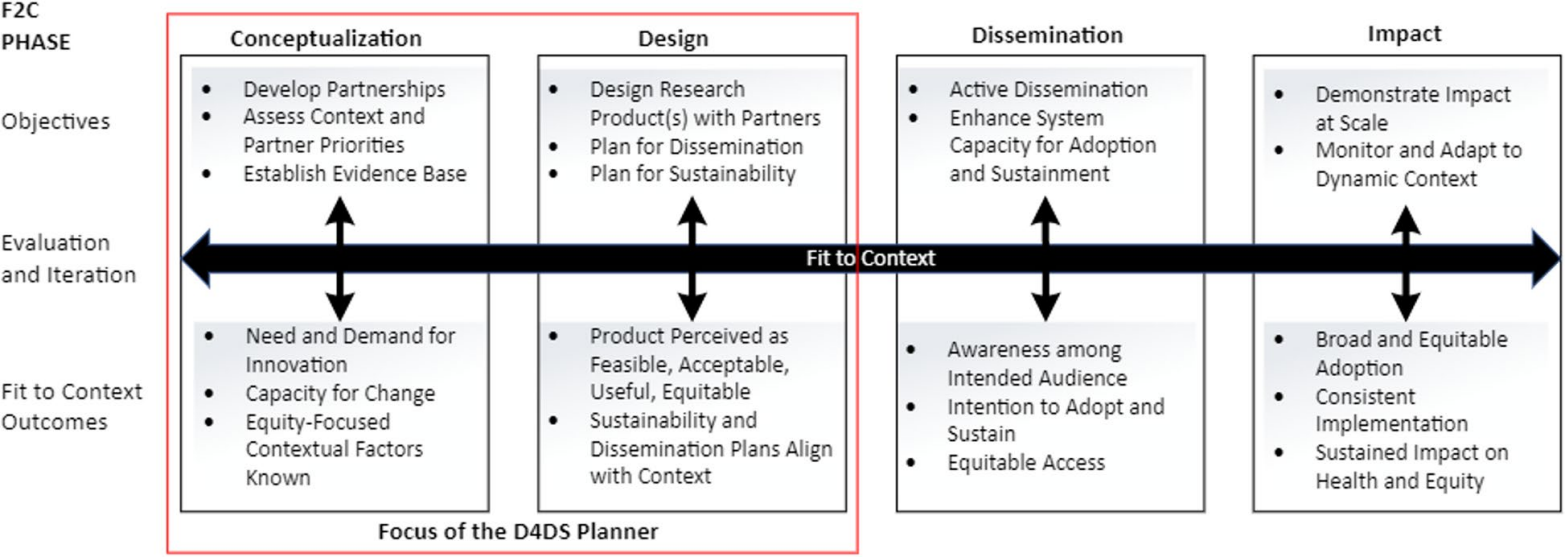
The Fit 2 Context (F2C) Framework for Designing for Dissemination and Sustainability

The F2C process framework for D4DS aims to support research product adoption, sustainment, and equitable health impacts. The framework includes four phases: conceptualization, design, dissemination, and impact (Fig. 1) [5]. In the first phase, conceptualization, the objectives are to establish partnerships, identify relevant evidence, and assess the context, in order to establish need and demand for an innovation as well as capacity for change. The second phase, design, includes co-design with users of an innovation (including packaging for use in real-world settings) and dissemination and sustainability plans. In D4DS the responsibility for active dissemination, the third phase, is positioned within the research team and their partners, institutions, and funders. Active dissemination involves deploying the dissemination plan, which specifies the intended audience (who you are trying to reach), the message (what information or product is being disseminated), and the communication channel (how and by whom the message will be shared) [8]. The fourth and final phase, impact, examines research product adoption, implementation, sustainment, and broader impact on health and health equity [5]. As a new process framework to guide D4DS, the F2C framework would benefit from tools to help guide its use and application in D&I research, as other D&I frameworks have done successfully [12–15]. For this first version of the tool, we focus on guidance for research teams and clinical and public health practitioner partners for use in operationalizing the F2C conceptualization and design phases.

Health equity is defined as having a fair and just opportunity for all people to be as healthy as possible [16]. We expect that D4DS principles could help promote health equity by engaging historically marginalized populations in developing effective interventions [17, 18] and products that meet the needs of these potential users and using strategies (e.g., develop local policy, using technology) that make effective interventions equally accessible in their contexts to generate equal opportunities for health [19, 20]. This may only be realized if the D4DS process is conducted using equity principles (e.g., including the marginalized population, gatekeepers, and diverse groups in developing your product, dissemination, and sustainability plans; using principles of reflexivity and trust building to engage meaningfully) [21–23]. We acknowledge that D4DS may not address the scope of obstacles historically marginalized populations face, such as poverty, discrimination, powerlessness, quality education and housing, yet, meaningfully engaging historically marginalized populations in product design and dissemination and sustainability planning is hypothesized in the F2C framework to impact health equity [24–28]. The F2C framework includes equity-oriented outcomes at each phase—including assessing equity-focused contextual factors in the conceptualization stage and engaging partners to design products that are feasible, sustainable, effective, and equitable in real-world settings in the design phase, the two phases that are represented in the D4DS planner.

To design the D4DS Planner, we engaged potential users using user-centered design methodology. User-centered design provides methods for eliciting user perspectives, preferences and ideas to co-design technologies [29]. This methodology is based on the premise that users are significant and useful partners in the knowledge-production and development process [30, 31]. User-centered design has been increasingly used as a method for the design of health technologies to empower users by involving them in the development and to ensure tools are more likely to be engaging and effective [30, 32, 33]. This paper describes the development of the D4DS planner via user-centered design methods, its features and functions, the usability testing of the platform, and future directions for the D4DS planner.

## Methods

### Purpose and user-centered design overview

We used user-centered design methods to develop a web-based interactive tool to support researcher application of D4DS principles and apply the F2C framework in planning and conducting D&I research. Specifically focused on the F2C conceptualization and design phases, the tool supports completion of 7 action items. Conceptualization phase action items include identifying and engaging with partners representing adopter and influencer groups, articulating the problem to be solved from the partners’ perspective, and assessing the context for intended use of a product that aligns with the problem to be solved. Design phase action items include co-designing the product, a dissemination plan, and a sustainability plan with partners. An overarching action item is iterative evaluation of “fit to context” outcomes at each phase.

**Fig. 2:**
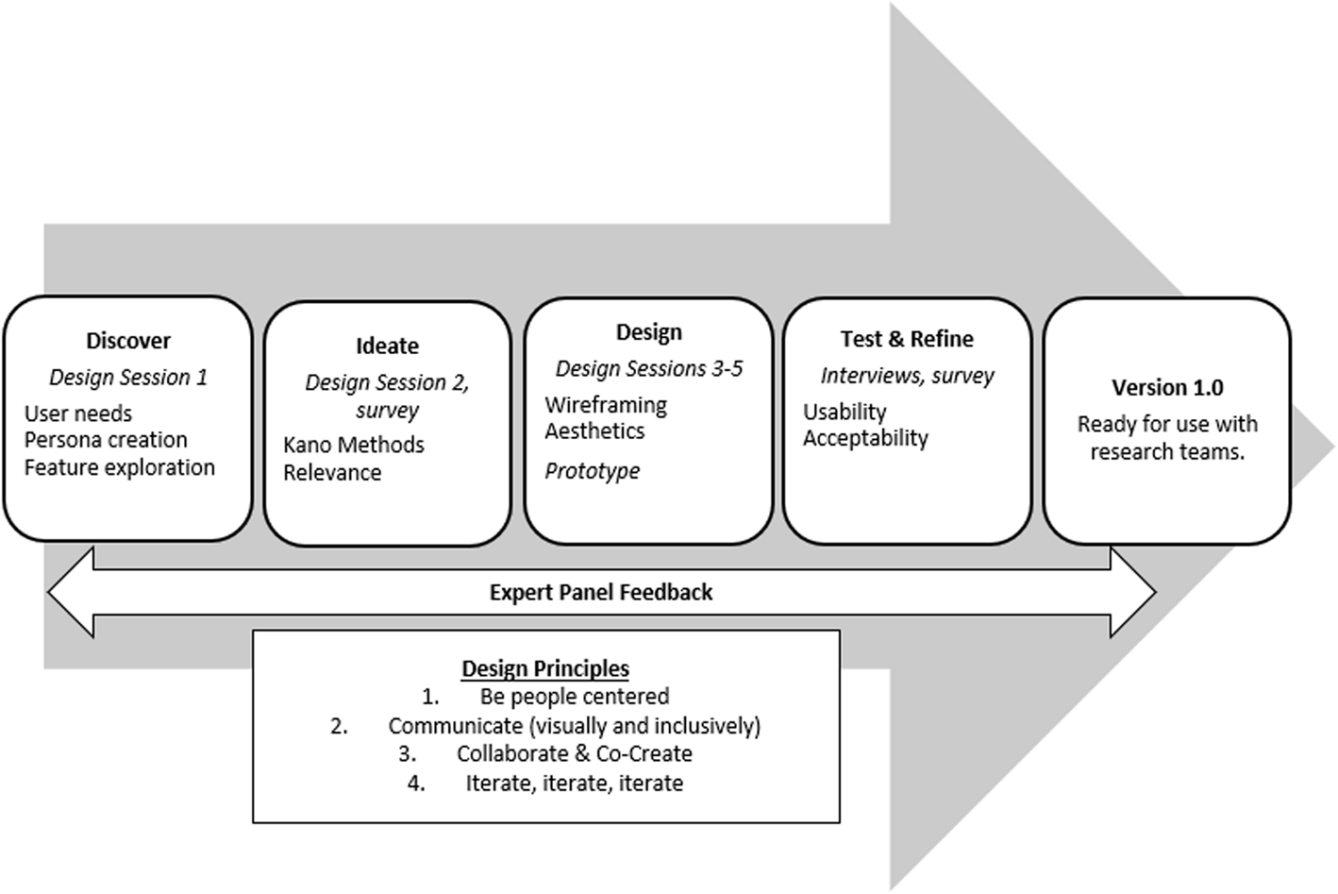
User-centered design process for the D4DS planner

A user-centered design approach engaged users and an expert panel in a mixed methods iterative user-centered design process starting from discovery to design and testing (Fig. 2). Qualitative and quantitative data were collected simultaneously during design sessions and merged to understand end-user's needs and desires for the tool. We utilized well-established user-centered design principles throughout this process that focused on: 1) being person centered; 2) communicating visually and inclusively; 3) collaborating and co-creating; and 4) iterating [34, 35]. Facilitators were intentional about engaging co-designers using equity centered principles such as using clear and easy to understand language, providing equal opportunity for sharing, and respecting and valuing all ideas [17]. We conducted five 90-min design sessions over Zoom from September 2022 to February 2023 (Table 1). Design session results were used to create a working-version prototype tool that was tested for usability and refined.

**Table 1:** Overview of design process and results

Exercise	Description	Overall Design Impact
**Design Session 1**		
1.1	how the tool could benefit and empower users	Goal of tool: Increase the transparency of D4DS and accessibility to multiple audiences, and foster collaboration Primary users of the tool: researchers (with various level of D&I knowledge/background) 35 potential features
1.2	who might benefit from using this tool	
1.3	persona creation^a^ and value proposition generation exercise	
1.4	Free listing possible features	
1.5	Prioritizing features	
**Design Session 2**		
2.1	Kano Methodology^b^	13 features selected Key content areas: dissemination; education/literature; grant language; partner engagement; sustainability planning; methods
2.2	free list any other feature ideas and identify their favorite feature from the Kano survey	
2.3	grouped features into content areas for learning	
2.4	prioritized the most important content areas	
**Design Sessions 3–5**		
	iteratively envisioning and creating wireframes of the features that were prioritized	Wireframes for 1) account creation, 2) a questionnaire that would allow users to set-up a project and guide them on how best to use the tool, 3) a roadmap or visual process that walks the users through the D4DS process, 4) and the landing page

^a^Persona creation is defining different user types that might use our tool and how they may interact and benefit from the tool

^b^Kano Methodology is a survey used to prioritize potential features [40, 41]. The model classifies features as Must-have (i.e., I expect it and would be dissatisfied without it), One-dimensional (i.e., I expect it), Attractive (i.e., I like it), and Indifferent (i.e., I’m neutral)

### Participants and recruitment

Users included researchers with experience conducting a health-related project, including academics (e.g., researchers, students, project managers) and clinical and public health practitioners (e.g., clinicians, health department employees). Using purposive and snowball sampling, we recruited 14 users (71% academics and 29% practitioners) to participate as co-designers. Email invitations sent out to users included a brief description of the project goals and D4DS principles. Each co-designer was asked to participate in at least two design sessions. Co-designers were from varying disciplines including public health, genetics, design, health communication, and social work and reported varying levels of D&I knowledge prior to starting the design sessions, with 21% (*n* = 3) reporting below average, 43% (*n* = 6) reporting average, 36% (*n*=5) reported above average knowledge.

The expert panel consisted of academic faculty members (*n* = 6) who are leaders in D&I science, including the developers of the F2C framework, across three academic institutions. Collectively, the expert panel focuses on public health research in both clinical and community settings and has expertise in the development, implementation, and evaluation of digital health tools. The expert panel members were engaged separately from users in a subset of the design exercises and usability testing.

### Data collection and analyses

All design sessions were conducted using Mural [36] or Figma [37], collaborative platforms that allowed users to contribute written artifacts (i.e., responses) during the session. FigJam, a tool in Figma, was used to draw collaboratively during design sessions using an online whiteboard. Following each design session, qualitative data were summarized and merged with written artifacts (e.g., a sticky note contributed on the mural board) from design sessions in a written report. The Kano Model of Customer Satisfaction survey, a valid and internally reliable (Chronbach’s alpha of 0.7) instrument, [38, 39] was used within the second design session to collect quantitative data to prioritize features [40, 41]. Kano survey results were analyzed following the design session in Excel using published methods for scoring features as must-have, one di-mensional, attractive or indifferent [40, 41]. Weekly team meetings were used to review the written report and survey findings to reach consensus on design impacts (i.e., how we would incorporate user feedback into design features of the D4DS planner).Once a working-version prototype tool was created, usability testing was conducted with a separate group of users who were not familiar with the tool and expert panel members using a combined think-aloud (qualitative) and survey-based approach using the 10 item System Usability Scale (SUS) and five items assessing perceived usefulness and appropriateness, both reported on a 5-point Likert scale. The SUS is a widely used scale with acceptable levels of reliability (coefficient alpha of 0.91) [42]. The think-aloud qualitative data was analyzed using an affinity grouping exercise conducted by four team members (MMK, TS, AL, BM) [43, 44]. Survey data were analyzed in Excel with SUS data using a published methodology that generates a score ranging from 0 to 100 and a descriptive mean scores generated for usefulness and appropriateness of the tool [45]. The prototype was revised and usability was re-assessed by the same users.

The timeline and further details on the methods for data collection, analysis and results for each design session and usability testing are presented below in the Design process and results section. The project was approved as an exempt study by the Washington University in St. Louis Institutional Review Board (#202207165).

### Design process and results

#### Design session 1: Discover

The first design session engaged 6 co-designers to identify the need and demand for key issues this tool should address and explore potential features that will address these needs. We started this design session with a brief overview of D4DS principles and then conducted five design exercises (DE). Co-designers started the session by adding sticky notes to free list: 1) how the tool could benefit and empower users (DE 1.1) and 2) who might benefit from using this tool (DE 1.2). Next, we conducted a persona creation and value proposition generation exercise (DE 1.3). Personas are archetypes of different users who could use the tool and has been used in design to ensure diverse perspectives are accounted for in product design. Therefore, in our sessions, we asked co-designers to consider the perspectives of other potential users (listed in DE 1.2) using a template value proposition stated (i.e., I’m a (user type), who uses the D4DS web tool to (use case) to define (impact)). We then asked co-designers to free list potential features (i.e., ways a user may interact with the content and experience learning in the tool (DE 1.4). Free listing features is a fast way to generate many ideas in a short period of time [46]. Lastly, co-designers placed the listed features into a prioritization bullseye and discussed their ideas by giving 2 min presentations on their favorite ideas (DE 1.5).

#### Results: Design session 1

The overall design impact of the first design session was that the tool should increase the transparency of D4DS and accessibility to multiple audiences, and foster collaboration among the research team and community partners. The tool is intended to help users plan their research and have D4DS as a key principle in their research from the start. To provide education, users suggested the tool contain a repository of key references and resources for individuals engaging in D4DS work. Users felt the tool should provide methods and strategies for engaging community partners in D4DS work. When asked who would benefit from the tool (DE 1.2), there was an emphasis on providing resources not only for people with D&I background, but also the people they partner with, whether that’s a site champion, an individual implementing an intervention, or a software developer designing materials for dissemination. While co-designers felt the primary users were researchers (with various level of D&I knowledge/background), they felt strongly that the tool would facilitate conversation with other partners. Other partners may include anyone who may influence the uptake of the product including community members and organizations, practitioners (e.g., clinicians, public health), policy makers, commercial partners, and funders. The main impact of the tool (DE 1.3) was described as: 1) enhancing co-design processes, as well as dissemination and implementation research; 2) increasing understanding of D4DS for multiple audiences; and 3) improving the design, effectiveness, and sustainability of research products. The users free-listed (DE 1.4) 35 features that were grouped into five categories: grant proposal resources, methods/tools, dissemination resources, educational materials, and general format ideas. The research team combined similar features to generate a final list of 25 features (Table 2), which was used as input for the next design session to prioritize features.

**Table 2:** Kano survey results

Classification^a^	Feature/Functionality^b^
**Must-have** A user expects this feature to be implemented and they would be dissatisfied if the feature was not available	1. **prominent links to other resources**
**Attractive** A user may or not expect this feature but it would make them satisfied if it is implemented	**2. a step-by-step guide to applying D4DS (aka roadmap)** **3. a questionnaire that guides users to the content** **4. template of language for funding proposals** **5. a template of how best to engage with partners** **6. a fillable form of a research plan** **7. methods at each stage of the research process** **8. an account to save a user’s work** **9. search for examples of grant section, case studies** **10. customizable figure of a common model/theory/framework** 11. search methods within topics and each stage of the design process 12. allows users to contribute content (e.g., case examples) 13. testimonials about users’ experiences 14. a blank roadmap that could be used during partner engagement 15. a tab specifically for community partners 16. provides images/icons for infographic development 17. an interactive social network map of users of this tool
**Indifferent** A user has a neutral opinion about whether a feature is implemented	18. short video presentations showing engagement methods or examples **19. opportunities to provide feedback via a ‘contact us’ interface** **20. has a large list of dissemination and sustainability literature** **21. allow users to search a database for references/literature by topic** 22. its twitter account so users can interact with developers of the web tool and with other users 23. an embedded twitter account 25. opportunities to provide feedback via a pop-up survey

^a^There were no features classified as one-dimensional, therefore, it is not represented in the table

^b^bolded items indicates those that were ultimately included in the tool

#### Design session 2: Ideate

The second design session focused on further exploring and prioritizing features. Using information from design session 1, we utilized the Kano Model of Customer Satisfaction exercise for prioritization of the 25 features (Table 2) [40, 41]. The Kano Model classifies features as Must-have (i.e., I expect it and would be dissatisfied without it), One-dimensional (i.e., I expect it), Attractive (i.e., I like it), and Indifferent (i.e., I’m neutral). Co-designers and expert panel members completed the Kano as an electronic (REDCap) quantitative survey during or outside the session (DE 2.1) [40]. After completing the Kano survey, the 3 co-designers performed an exercise to free list any other feature ideas and identified their favorite feature from the Kano survey (DE 2.2). Co-designers then grouped features into content areas for learning (DE 2.3) and prioritized the most important content areas to identify which ideas are most important to users (DE 2.4).

#### Results: Design session 2

A total of 17 participants (24% practitioners) completed the Kano survey. Table 2 lists the features in order of prioritization according to the Kano survey responses. We identified 1 Must-have feature, 17 Attractive features, and 7 Indifferent features. The Must-have feature was URL links to other content that falls within the discipline/field of D4DS, especially dissemination and sustainability resources. The number of Attractive features was a promising finding given the many potential opportunities to delight users (a key goal of design), with a relative low risk of dissatisfaction if the feature was not integrated at all. Indifferent features were defined as those which would not yield either satisfaction or dissatisfaction for our users. As a result, the Indifferent features (e.g., an embedded twitter account, short videos showing engagement methods or examples, a pop-up feedback survey) occupied the bottom of our development prioritization, and most were ultimately not incorporated into version 1.0 of the web tool.

#### Design sessions 3–5: Wireframing

The next 3 design sessions focused on iteratively envisioning and creating wireframes of the features that were prioritized in the previous design session. Wireframes are illustrations of a product that are not yet built and typically lack functionality but represent the interface and its intended features and functionalities. The iterative design process allowed users to draw and give feedback on design options (e.g., wireframes) in successive versions. During design session 3, 4 co-designers ideated and drew 1) account creation, 2) a questionnaire that would allow users to set-up a project and guide them on how best to use the tool, 3) a roadmap or visual process that walks the users through the D4DS process, 4) and the landing page. In design session 4, 4 co-designers provided feedback on two versions of the roadmap and sketched a final version of the roadmap. We also discussed which features co-designers wanted to be present on each page that derives from the roadmap and sketched the layout of a single page. In design session 5, co-designers provided comments on a refined version of the single page sketch, sketched the layout of the education hub and spent time discussing the name of the tool and each feature.

#### Results: Design sessions 3–5

**Fig. 3:**
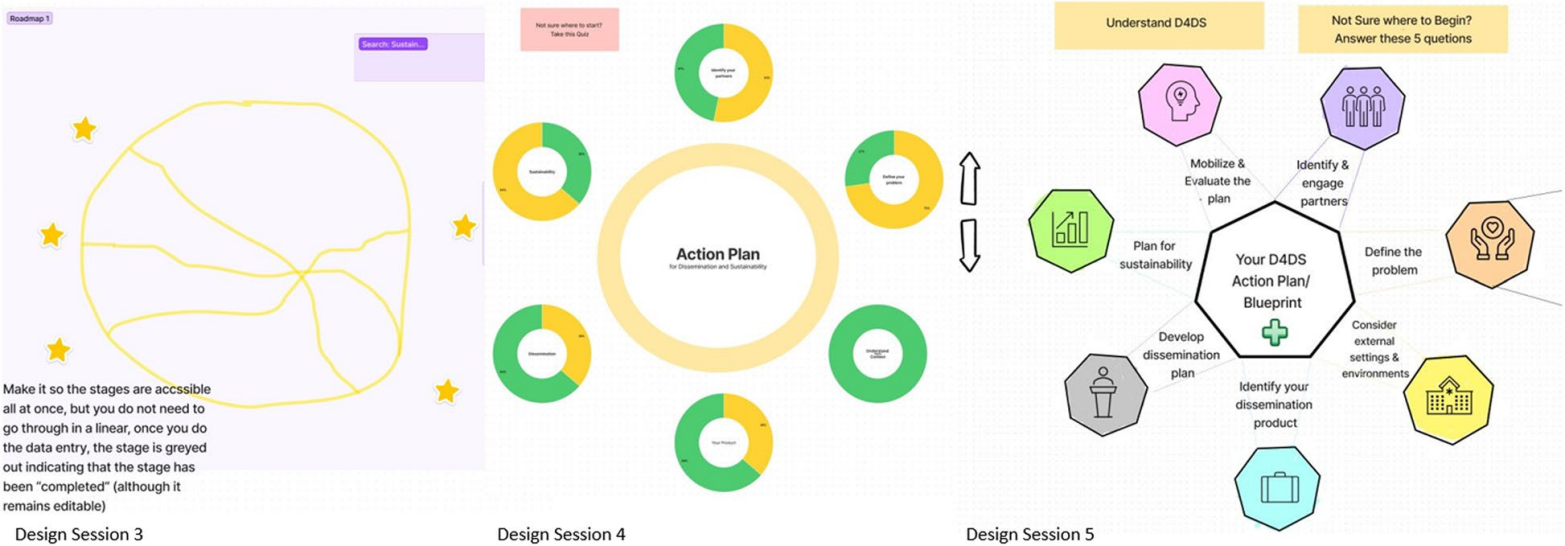
Iteration of roadmap wireframe from design sessions 3-5

Co-designers felt that users should have the option to use the tool as a guest or with an account, making it clear to users the benefits of having a login (e.g., saving data, returning later to update work). Discussion identified that the login should be simple but make users feel that the information they add to the tool is secure. A major focus of our prototyping sessions was brainstorming and drawing the feature of a roadmap that would help users walk through a D4DS process. The wireframe of the roadmap was iterated in each session (Fig. 3), with co-designers realizing they did not want this to feel like a linear process. Co-designers were critical in the process of developing easy to understand, action-oriented language to name different features of the tool (e.g., D4DS Planner, action planner, action item, cue to equity). The content for each feature was developed by our team and the expert panel based on published literature.

#### Usability testing: D4DS planner prototype

Following all design sessions, our team delivered low-fidelity wireframes and features to a software development company (HICAPPS, https://hicapps.com) that has expertise in developing health-related tools. Our team collaborated with HICAPPS on an iterative build of a prototype of the D4DS Planner to ensure that all feedback from design sessions was incorporated.

Usability testing on the prototype tool was conducted with a separate group of 11 users (55% academics and 45% clinical and public health practitioners) who were not familiar with the tool using a combined think-aloud and survey-based approach. Five members from the expert panel assembled at the start of the design process also participated in the think-aloud testing only. These test users were asked to use the D4DS web tool on a laptop computer in a private space in-person or via Zoom to conduct the think-aloud testing. The test users were given a general description of the web tool but were not given explicit instructions on how the web tool operated or how it was designed to be used for developing a dissemination and sustainability plan. Test users were asked to carry out two out of a possible three heuristic tasks, including: 1) familiarize themselves with the web tool and figure out the purpose and function of the web tool, 2) create an account, and 3) set up a project to create a D4DS action plan.

To assess the heuristic usability data, an affinity grouping [43, 44] exercise was conducted in Mural, an online collaboration software app, by four team members (MMK, BM, TS, AL) who were integrated into the design and development of the web tool. The research team analyzed internal notes taken during the usability sessions and re-watched recordings of users navigating the tool. Summary phrases were extracted and typed on Mural’s “sticky notes” and mapped together based on similarities in relation to four categories and the collaborator type, e.g., researchers, practitioners, and leadership. The four categories were as follows (1) What works well in the D4DS web tool? (2) What is not working well, or what are the significant “pain points,” in the D4DS web tool? (3) What changes do users want to see in the D4DS web tool? (4) Other comments that pertain to the usability of the D4DS web tool. At the end of the usability sessions, test users completed the SUS to measure perceived usability of the tool quantitatively [47]. Usability results were used to refine the prototype tool. The refined version of the D4DS planner was retested among the same users who participated in the first round of usability testing. During this round of testing, five questions (reported on a 5-point Likert scale) about the tool's usefulness and appropriateness were added to the survey.

## Results

### Usability results

The affinity grouping synthesis of qualitative usability data is presented in Table 3. Overall, users across the three groups (academics, practitioners, and experts) expressed that the prototype web tool had a professional “look and feel” and contained action-oriented content not often considered when planning for dissemination and sustainability. Users scored the prototype tool a 66 using the SUS, which is slightly below the standard cut-point of 68 to indicate above average usability [48]. While the web tool contained much high-level content, some users felt that there were too many words on the screen. Our main challenge was to provide the user with enough information, while not over-burdening the user who might become reluctant to use the web tool because of its relative complexity. In general, these results were helpful in allowing our team to identify areas of the prototype web tool that needed to be revised to achieve a balance of providing guidance and not overly saturating the interface with directions and jargon. We curated a list of changes that were communicated to the developers of the web tool (Supplementary Material 2) and made several wording changes to the content to reduce complexity and breadth. Some of the major changes included: 1) the addition of instructional videos and a guided tour that show users the key features of the tool upon first logging in; 2) reduction of text and simplifying language; and 3) simplification of the login and project set-up. Following these updates, usability was reassessed on the first version of the D4DS Planner (presented below) by 7 of the 11 users (57% academics and 43% practitioners) who participated in the first round of usability testing. After refinements, usability scored 71, which was a 5-point increase in the score moving the tool to be perceived as above average for usability. Overall, users felt the tool was useful and appropriate with a mean score of 4.1 (SD 1.1; Table 4).

**Table 3:** Summary of think-aloud usability testing results

Prompt	Academics	Practitioners	Experts
What works well in the D4DS web tool?	Academics liked that the D4DS Planner was action oriented and flexible to meet their project needs. Academics felt supported in this process by key features in the tool (e.g., key definitions, guidance questionnaires)	Practitioners liked how clear it was to use the different components of the tool including the account creation/login, navigating the landing page and action planner, and project creation. The clarity was enhanced by the clear instructions on how to use the action planner and ease of finding information. Practitioners also liked how the web tool is visually appealing	Users liked the visual aesthetic of the tool, felt it was organized in a logical manner, and believed there were good resources that supported the user in how to use the web tool
What are the significant pain points in the D4DS web tool?	Locating features and clarity on where to go within the D4DS tool was challenging. Setting up the project required additional clarity and parsimony	The three major pain points for practitioners were the delay in receiving the verification email, difficulty in answering some questions in the project set up (i.e. budget, main objectives) and that some parts of the tool were wordy (i.e. guidance)	The layout of the pages can be challenging because the user does not know to scroll down. There is tension between layperson language and jargon. There is too much language in general throughout the web tool, although users asked for directions to guide their interactions
What changes do users want to see in the D4DS web tool?	More guidance, tutorials, and examples to support users with get started and how to use the tool were requested. The use of simple language and project set-up requirements were a priority	More information about setting up an account and project is needed. Reducing the amount of wording in the guidance section of the tool could enhance the readability	Many of the changes have to do with the content or the jargon of the field. This presents a challenge. Because it is a specialized field and the point of the tool is to teach the language of the field, in addition to planning for projects
General usability comments	The D4DS Planner pushes teams to think critically about aspects that are not commonly considered from the early stages of a project (dissemination and sustainability)	The tool seems more tailored to academics than practitioners with regards to its perceived use and functionality (e.g. account set up). Practitioners tend to be working with already designed products to implement and sustain, however the tool seems to provide support more towards designing a new product. The tool could also skip the email verification process like other web tools	Users seemed to engage with the tool easily and noted that the web tool was visually appealing and professional looking

**Table 4:** Perceived usefulness and appropriateness of the D4DS Planner

Question^a^	Mean(SD) (*n* = 7)
The information the tool provides is useful	4.3 (0.8)
The tool seems possible to use in my work	4.4 (0.8)
The tool would help my work be more impactful	4.1 (0.7)
The tool would help my work to reach a more diverse group of people	4.0 (0.8)
The tool would make the information I want easier to access	3.7 (1.1)
Overall score	4.1 (1.1)

^a^Questions were asked on a 5-point Likert scale (strongly disagree to strongly agree)

### The D4DS Planner

**Fig. 4:**
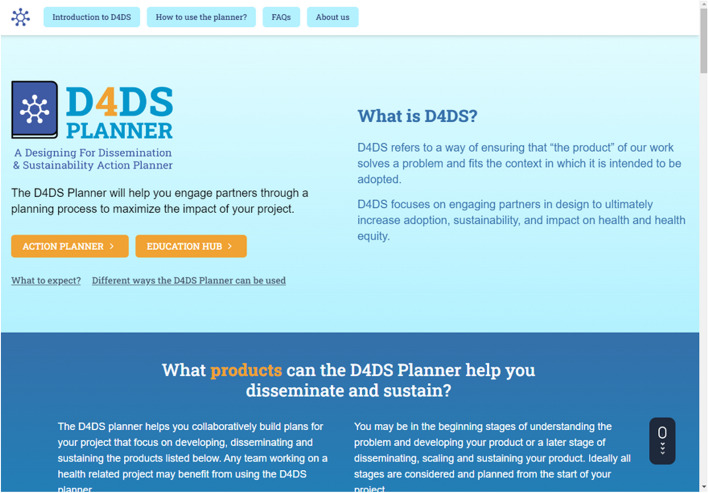
D4DS planner home page

The D4DS Planner is a digital platform designed to help users collaboratively apply D4DS principles and methods to a research project to maximize the potential of their work. The D4DS Planner has two main components: 1) the Education Hub (i.e., a searchable platform with literature, videos, websites, etc.) and 2) the Action Planner that can be accessed from the homepage (Fig. 4; d4dsplanner.com).

The Education Hub is available to users without logging in and includes resources (e.g., websites, journal articles, presentations and videos) to educate users on D4DS principles and methods. The Education Hub has search and filter functionality that allows users to find materials related to each D4DS step (called action items) and materials that provide methods, case examples and cues to increase focus on equity. The Action Planner allows users to interact and collaborate with a team in real-time or asynchronously on their project. Users are required to create an account before setting up a project so that all information is saved and can be downloaded as a D4DS Action Plan. The D4DS Action Plan is a detailed document outlining users' input from each activity that can be used to support D4DS and the application of the F2C framework. Supplementary Material 1 provides an example of a D4DS Action plan. It includes information specific to the team’s project that can be used to develop a grant proposal, communicate the value of your work, and justify funding.

**Fig. 5:**
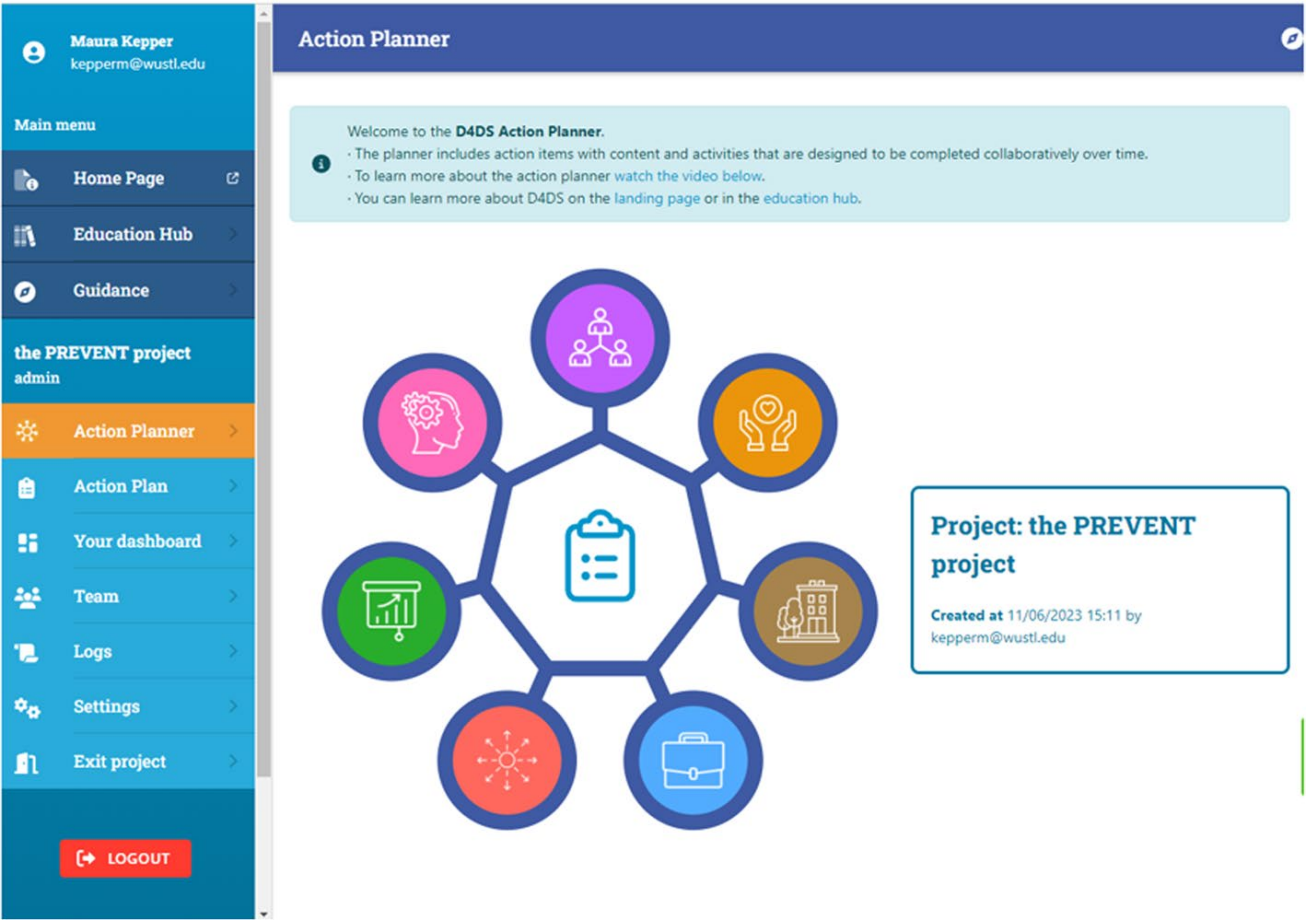
D4DS action planner

The Action Planner (Fig. 5) is composed of 7 “Action Items” based on the F2C framework’s conceptualization and design phases (Image 1 missing hyperlink) that guide users through the D4DS process.(5) Each A action Item includes an instructional video, interactive activities, educational content, and cues to equity. The cue to equity section prompts users to be intentional about how they are conducting their D4DS activities with the goal of developing equitable products (e.g., those that meet the needs of marginalized populations) that can be equitably accessed by the intended population over time. Content for these cues was generated by our team and expert panel, pulling from published literature and principles in community engaged research and human centered design [17, 49–51]. Examples of cues to equity, include: 1) “Consider who is present and, perhaps most importantly, who is absent in your studies and research team;” 2) “be attentive as to how you ask the questions and attend to issues of power, and of the implicit and explicit assumptions of your questions;” and 3) “think about ways to share information that reflect the values and cultures of your partners.” Each cue is relevant to the activity the team may conduct within the action item and provides further educational materials to help them operationalize the cue.

**Fig. 6:**
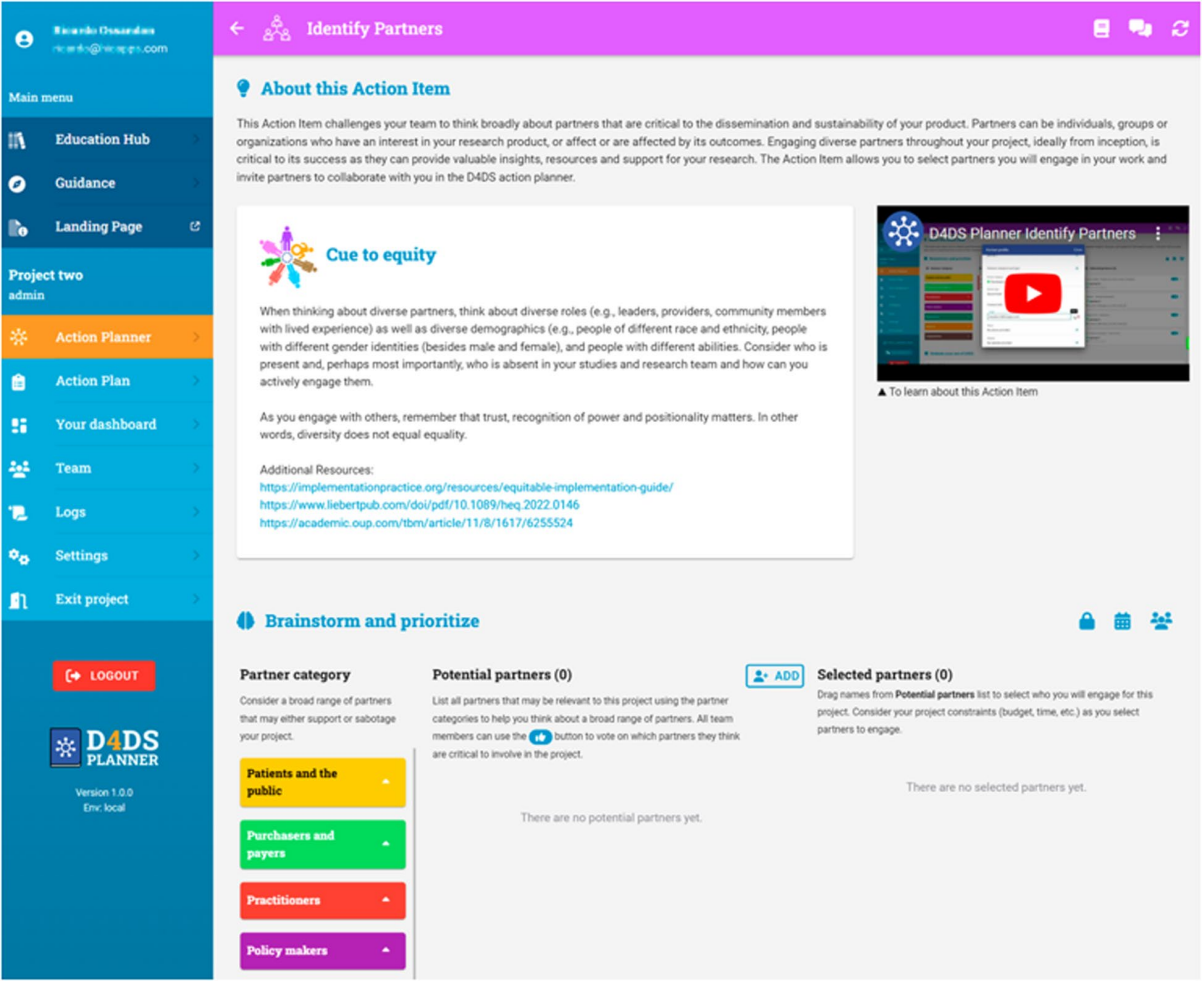
Identify partners action item in the D4DS Planner

Although the tool was built for researchers and research teams, critical to D4DS and strongly valued by co-designers were features that facilitated the engagement of community partners, including community members, implementers, public health organizations, policy makers, etc. The tool invites users to brainstorm and invite relevant partners in the “Identify Partners” Action Item (Fig. 6) to log-in and collaborate with them through the process. While these partners then become users of the tool in collaboration with the research team, these individuals were not included in our initial design process but will be engaged to inform future iterations. The tool includes features such as the ability to assign team members specific tasks with a due date and chat features that foster collaboration. As suggested by co-designers, this process does not need to be completed in it’s entirety or linearly, although some steps do build from others. The tool includes a guidance questionnaire that was co-designed to help users identify which action items are most relevant to their project. The 7 Action Items are:

- Identify Partners: This Action Item challenges users to think broadly about partners that are critical to the dissemination and sustainability of their product using the 7 P’s framework for stakeholder identification in outcomes and effectiveness research [52]. Partners can be individuals, groups or organizations who have an interest in the research product, or affect or are affected by its outcomes. As stated above, the Action Item allows you to select partners you will engage in your work and invite partners to collaborate with you in the D4DS action planner.
- Empathize and Outline the Problem: Users will engage partners to understand the problem from their perspective and generate a Value Proposition that clearly communicates how their product meets the needs of their target audience. In research, a value proposition can be used to communicate the value of our research to our partners, funding agencies, and the general public.
- Understand The Context: This Action Item includes key questions to help users think broadly about characteristics of the people, relationships, product, organization, and environment that may influence the ability to reach the target audience and sustain impact. The goal is to help users consider the multilevel nature of how context can impact how they share, adopt, use, and benefit from the product over time.
- Confirm and Co-design Your Product: This Action Item allows users to select methods for co-designing their product and packaging it for use in real-world settings. Using co-design methods to engage key partners in the design of a product increases the likelihood that your product will be perceived as feasible, acceptable, useful, and equitable.
- Develop a Dissemination Plan: This Action Item helps users generate a plan for how to share their product with key audiences, especially those outside of academia. In this action item, the team and their partners will brainstorm all the possible ways they may share about their product, prioritize which methods are the most feasible and will have the most impact, and generate a plan.
- Plan For Sustainability: This Action Item helps the team discuss and prioritize what is needed to sustain their product over time and create a practical action plan. The goal is for the team to think early and actively plan for sustainability to enhance the long-term impact of their work.
- Evaluate Iteratively: This Action Item helps users evaluate their use of D4DS and develop plans for evaluating dissemination and sustainability. In this Action Item, the tool helps users develop evaluation plans to maximize the intended impact of their dissemination and sustainability plans. Evaluation should be conducted iteratively, and users should return to this Action Item as needed to revise their plan.

## Discussion

The freely available D4DS Planner (d4dsplanner.com) is the first digital tool that supports transdisciplinary research teams in learning about and applying D4DS principles and the F2C framework in their work. Developed through a user-centered design process, the two main components of the tool are the Education Hub, which contains resources to help users learn about D4DS, and the Action Planner, to support the application of D4DS principles and the conceptualization and design phase objectives of the F2C framework.

User-centered design engaged primary users in co-designing the D4DS Planner that incorporated their experiences and met researchers’ needs and preferences [34]. Researchers indicated that the tool would be helpful in improving understanding of D4DS, enhancing co-design processes with partners, and ultimately would improve the effectiveness and sustainability of their products. Not surprisingly, features prioritized by the users were those that support learning about D4DS as this is a new and expanding area, including links to resources, and the process of engaging and collaborating with partners, including a roadmap tailored to a specified project to guide users in conducting D4DS. Overall, users liked the visual presentation of the tool and the actionable content that allows for developing a plan for dissemination and sustainability. The relative complexity in navigating the tool and having too many words in some sections were areas that users recommended modifying to enhance usability. While the user-centered design approach was time intensive, the content of the tool was new to many users and time was necessary to orient them to the content and solidify how our planner would be useful to end-users. Similarly to our learnings, Harrington et al., emphasized the importance of considering the history and context of the research environment and the user [53]. The inclusion of users throughout the prototype build phase may have been beneficial, rather than waiting until the end to gain feedback on usability. This more iterative approach may have saved us time and financial resources by reducing the number of changes necessary after usability testing.

The D4DS Planner web tool has several potential uses for health-related projects in different phases from conceptualization to sustainability. The aim of this tool is to support research teams to deepen their understanding of D4DS principles and methods and to facilitate their work as a team, along with their community partners, to identify and refine the solution (i.e., product). Additionally, this tool can aid in formulating comprehensive dissemination and sustainability plans with partner input. Early and ongoing planning for dissemination and sustainability enables the consideration of the intended audience and capacity and resources of the implementers and their contexts to enhance translation and utilization of research products in practice [2, 6, 54, 55]. Further, the Action Plan generated from the D4DS tool can support the development of grant proposals and grant dissemination plans.

Engaging community partners throughout the process of planning a research initiative is critical to ensure that products are designed to match contextual characteristics (i.e. priorities, needs, resources) of partners [5, 6, 56]. Community partners include anyone who may influence the uptake of the research product, such as patients and the public, practitioners, organizational leaders and policy makers. Users can invite partners to work with them in each step of the D4DS Planner by completing tasks synchronously during a meeting or asynchronously using interactive features that can administer questionnaires to community partners or assign specific tasks in the tool. Community engagement is critical to improving dissemination and sustainability by better aligning research activities with the priorities, needs, and assets of the intended users and local context [5, 49]. Several tools have been developed to guide conducting community-engaged research and the application of D&I frameworks; however, to our knowledge none have focused on engaging related to product development, dissemination, and sustainability [50, 57–61]. Engaging the community partners, particularly marginalized populations, in product design may promote health equity by developing interventions and products that meet the needs of historically marginalized populations and account for their context. Intentionally selecting partners that do not create, reinforce, or maintain existing inequities is important if the process is intended to promote health equity [62]. When done well, co-creating ways of sharing and sustaining the intervention may increase reach, adoption, and sustained impact among diverse populations and marginalized groups. There is need for equitable processes of engaging community partners in these activities to ensure the D4DS process results in intended equity outcomes (e.g., equitable access to the product, health equity). Based on input from our users, cues to equity were generated to support teams in using the interactive features of the tool and D4DS methods with intention and equity in mind, with the goal of improving dissemination and sustainability outcomes and impact.

### Limitations

The target users for the D4DS Planner are research teams conducting clinical and public health-related research. While our co-designers included academics and practitioners from varying disciplines and with varying D&I knowledge, we did not ensure diversity across demographics (e.g., gender, race/ethnicity, age). The tool is limited to the English language, which will limit the use of the tool globally. The tool was designed for users (academics and practitioners with experience conducting health-related projects/research) to engage community partners in the platform. While this tool provides a formalized process of engaging partners in D4DS and seeks to reduce barriers of engagement, it does not overcome the immense challenges of engaging community members, compensating them appropriately, and sustaining engagement over time with equitable involvement and input. Furthermore, we did not include community partners beyond practitioners or without research experience in our design sessions or usability testing. Usability testing was not conducted with collaborative teams (i.e., a researcher and community partner reviewing the tool together) which limited our ability to assess the tool’s features and functions that were designed to facilitate collaboration and engagement. Furthermore, while the idea for cues to equity to support teams with how they engage community partners originated from end-users, many with extensive experience addressing health equity, the content of the cues was not designed with an extensive group of community partners.

### Next steps

To evaluate the tool's impact, we will assess its reach and engagement using Google analytics (e.g., number of users who access the tool, duration of use, completion of tasks) and its effectiveness and context-dependent implementation through user case examples. We will also design studies to test whether facilitation strategies are needed to support use of the tool; with a particular focus on understanding whether research teams without a D&I scientist are able to effectively use and complete the tool without support from experts in the F2C framework, participatory co-design methods, and dissemination and sustainability planning.

The D4DS Planner is in its early iteration, and we are committed to refining the tool based on feedback from users representing diverse backgrounds, including more researchers in non-academic and low resource settings and community partners. These varied perspectives are crucial, aligning with the tool's primary goal of engaging a broad spectrum of partners in dissemination and sustainability planning to enhance product-context fit and ultimately promote health equity. Immediate future work will focus on usability testing among research teams, including community partners, and examine whether the tool was used as intended and/or differed by the type of user (e.g., practitioner, researcher, community partner) and the impacts of using this process. As the tool is used, our team intends to generate case examples of use and conduct follow-up questionnaires and interviews with research teams and the community partners engaged to understand their experience, perceptions of the tool, and impact on their work. There will be a focus on eliciting perspectives from community partners to refine and tailor the tool further to support engagement with community partners and facilitate the team-based approach. Specifically, we will seek to understand if community partners are able to participate longitudinally in an in-depth manner; the costs of this involvement from multiple perspectives; how they felt about engaging in dissemination and sustainability planning, specifically through the D4DS Planner, to refine features of the tool. Long-term, we plan to engage community partners in future design sessions that aim to expand the tool or create another version of this tool to include features that support health-related community work. Additionally, we will explore teams’ ability to easily use the cues to equity to engage partners and whether cues have the intended impact on how the D4DS process is conducted and on health equity outcomes. Furthermore, as a first step to expand access to diverse populations we will consider building a language translation feature into the tool.

## Conclusions

Dissemination and sustainment are critical to maximize the impact of research on health and well-being. The D4DS Planner can help research teams apply the F2C framework for D4DS, facilitate partner engagement in research initiatives, and enhance the overall impact of their work. To our knowledge, this is the first D&I tool that facilitates engaging partners in planning for product development, dissemination, and sustainability.

## Supplementary Information

Supplementary Material 1.

Supplementary Material 2.

## Data Availability

The datasets used and/or analyzed during the current study are available from the corresponding author on reasonable request.

## Abbreviations

AL: Allison L'Hotta PhD, OTR, OTR/L
BM: Brad Morse, PhD
D&I: Dissemination and Implementation
D4DS: Designing for Dissemination and Sustainability
DE: Design Exercises
F2C: Fit to Context framework
MMK: Maura M. Kepper, PhD
SUS: System Usability Scale
TS: Thembekile Shato, PhD, MPH
UCD: User Centered Design
