# Supplementary material for: Supporting teams with designing for dissemination and sustainability: the design, development, and usability of a digital interactive platform
Source: Implement Sci. 2024 Dec 31;19:82. doi: 10.1186/s13012-024-01410-7 (PMC11686880; doi:10.1186/s13012-024-01410-7)
Supplement: Supplementary file 1 — Supplementary Material 1. [file 13012_2024_1410_MOESM1_ESM.pdf]

D4DS Planner Action Plan

# Social isolation interventions

**What is the health problem you are trying to solve?**

Reducing social isolation among individuals with cognitive impairments to improve health and well-being.

**What is the product of this project you are trying to disseminate and sustain?**

Intervention (e.g. program, treatment, service)

## Constraints

Project budget: $200,000

Set project funds aside for dissemination and sustainability: I don't know

Generated At: 04/07/2024 14:45

# Identify Partners

These partners have been selected:

| Partner Name | Category (Type) | Contact Info |
| --- | --- | --- |
| Neurologist- Dr. X | Practitioners (Physicians/Nurse practitioners/ Physician assistants) | -- -- |
| Caregiver of a person with acquired brain injury | Patients and the public (Caregivers) | Email: email@email.com -- -- |
| Person with acquired cognitive impairment | Patients and the public (Patients (includes children and/or adults)) | Email: test@email.com -- -- |

## Evaluation Questions:

**Selected a diverse group of partners to engage?**

yes

**Considered who may be absent from your team and identified how to engage this group of individuals?**

yes

## Empathize and Outline the Problem

This is a draft of the Value Proposition Statement

**Our social engagement intervention helps adults with cognitive impairments who want to combat social isolation by improving patient identification and availability of resources and increasing social connections and overall well-being. This social engagement intervention is innovative because it provides personalized support**

**Evaluation Questions:**

**Developed an understanding of the problem you are trying to solve from the perspective of your partners?**
yes

**Generated a value proposition that communicates how your product specifically benefits your target audience and responds to barriers they face?**
yes

# Understand The Context

## Target Audience

| Question | Answer |
|---|---|
| Who are the main people/groups you are trying to reach (your target audience)? | Adults with cognitive impairments and their caregivers |
| What characteristics about your target audience may impact their ability to use and benefit from your product now and in the future? Consider issues such as history, beliefs, and relationships, as well as their age, gender, culture, preferences. | Presence of cognitive impairments, reduced intrinsic motivation, reduced problem solving skills |

## The Product

| Question | Answer |
|---|---|
| What are the features of this 'product' that meet the needs of your target audience? | supports social engagement, helps with problem solving skills |
| What kinds of changes or adaptations (e.g., change content, format, and/or delivery methods) may you need to make to the product to help it continue to be used in your context (support sustainment)? | may need to make it a less complex intervention, need to support virtual administration of intervention |

## Implementers

| Question | Answer |
|---|---|
| Who are the people who will be delivering and using the product (the implementers)? | Clinicians, community organizations |
| What characteristics about the implementers may impact their ability and willingness to use and benefit from your product now and in the future? Consider their beliefs, age, gender, culture, preferences and competing demands for implementing the product. | time, knowledge/skills, awareness of the intervention |

## Other People

| Question | Answer |
| --- | --- |
| What other people (beyond implementers) may influence your product's ability to reach and impact your target audience now and in the future (support sustainment)? | patient, caregivers |
| What characteristics about these other people may impact the ability of your target audience to use and benefit from the product? | buy-in, time, cognitive skills, social support |

## Organization and Setting (Inner Context)

| Question | Answer |
| --- | --- |
| What factors are influencing the organization's readiness to try out, apply, and continue use of the product? | people to implement the intervention |
| What resources are needed to use and sustain the product over time? | personnel, technology, money |

## Big Picture/External Factors (Outer Context)

| Question | Answer |
| --- | --- |
| What policies or regulations may influence the reach and long-term use (sustainment) of your product? | No answered yet. |
| What social and cultural norms may influence the reach and long-term use (sustainment) of your product? | No answered yet. |

# Context Chart

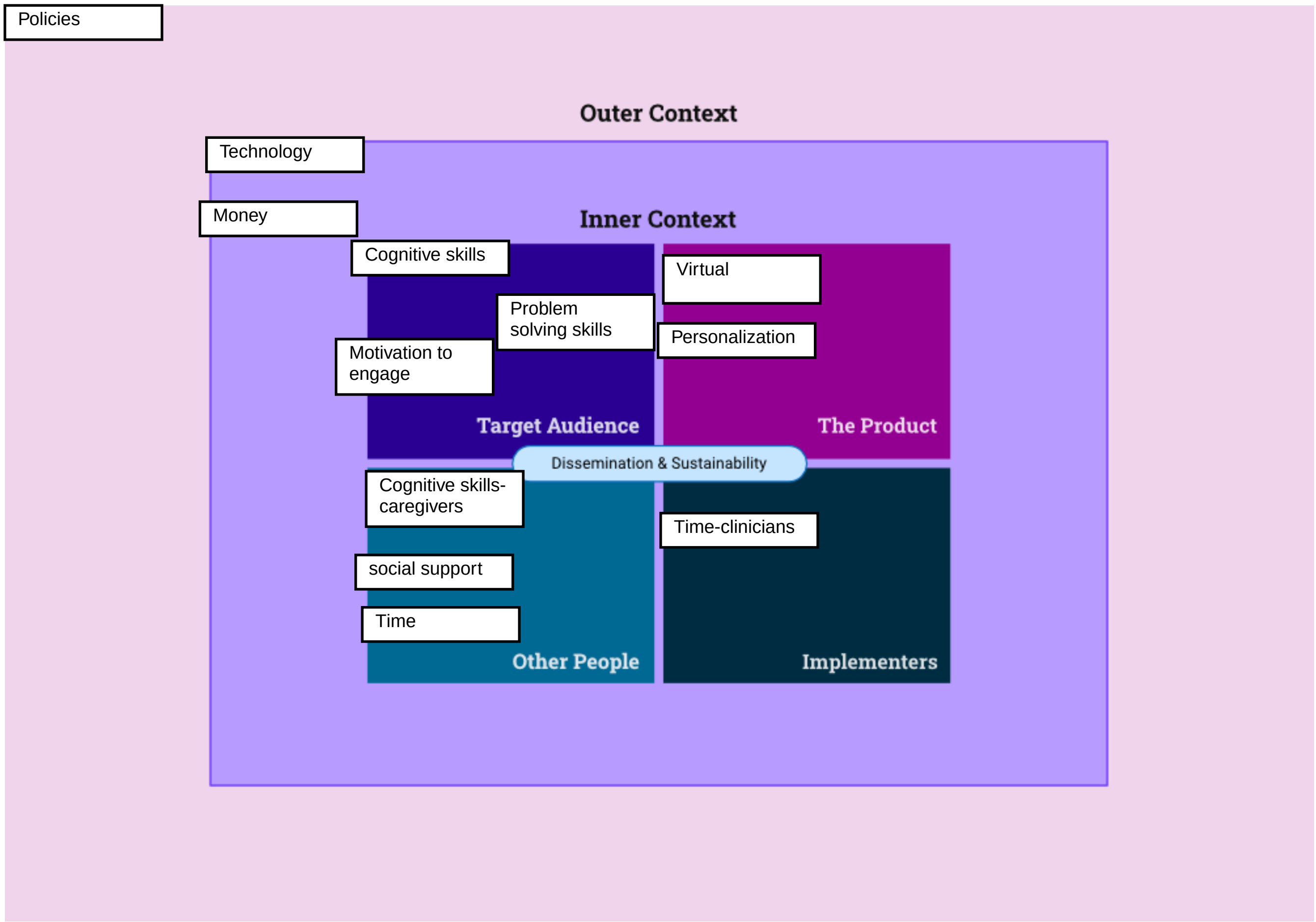


## Evaluation Questions:

**Considered keys group's (i.e., target audience, implementers) capacity for change?**
yes

**Accounted for characteristics in the setting and broader social systems that may influence your ability to share and use your product long-term?**
yes

# Confirm and Co-design Your Product

## Selected Co-Design Product:

### Intervention (e.g., programs, treatments, services)

| Selected Methods |
| --- |
| Experience-based co-design |

## How to use the selected Co-Desing methods

## Experience-based co-design

**What team member(s) has experience or expertise with this method?**
No team members selected.

**What partners will you involve in the co-design process?**

| Category | Type | Name |
| --- | --- | --- |
| Patients and the public | Caregivers | Caregiver of a person with acquired brain injury |
| Practitioners | Mental health providers (psychiatrists, psychologists) | Psychologist |

**What are your key benchmarks for success in using this co-design method? This may include a timeline, engaging diverse partners, learning a new method, etc.**

**Learning steps in the co-design process. Completing preliminary co-design of intervention in 8-12 months.**

**What resources may you need to support your use of this co-design method? Consider funding, time, personnel, etc.**

**Methodologist, funding, time**

## Evaluation Questions:

**Made decisions about what co-design method(s) to use that gather input from key partners?**
yes

**Selected co-design methods that are feasible for your team to achieve?**
yes

# Develop Dissemination Plan

| Dissemination methods | Target audience category | Type of people to reach |
|---|---|---|
| Podcast | Patients and the public | Caregivers |
| Meeting (in-person/virtual) | Practitioners | Mental health providers (psychiatrists, psychologists) |

### Podcast

| Question | Answer | Date |
|---|---|---|
| What is the intended impact of sharing this? | Raise awareness or educate your audience, Impact health and equity, Equitable access to my product | 04/07/2024 |
| Where will you share this? | Online | 04/07/2024 |
| How much will it cost to share it? | | 04/07/2024 |
| Who will develop it? | Team | 04/07/2024 |
| Who will share it? | Podcast group | 04/07/2024 |
| When will it be shared? | Not answered yet | Not answered yet |
| What are the resources you still need? | Funding, People, Skills, Technology | 04/07/2024 |
| Use this space to detail assignments and tasks | HL- identify target podcast by 1/1/25<br>AR- develop question for podcast by 2/1/25 | 04/07/2024 |

### Meeting (in-person/virtual)

| Question | Answer | Date |
|---|---|---|
| What is the intended impact of sharing this? | Raise awareness or educate your audience | 04/07/2024 |
| Where will you share this? | Online meeting | 04/07/2024 |
| How much will it cost to share it? | Free | 04/07/2024 |
| Who will develop it? | Team lead | 04/07/2024 |
| Who will share it? | Team lead | 04/07/2024 |
| When will it be shared? | Not answered yet | Not answered yet |
| What are the resources you still need? | People, Technology | 04/07/2024 |
| Use this space to detail assignments and tasks | AR- Coordinate meeting with practitioners by 11/1/24 | 04/07/2024 |

## Evaluation Questions:

**Made decisions about what dissemination methods to prioritize in collaboration with key partners?**
no

**Selected dissemination methods that will maximize reach and impact for your target audience(s)?**
no

**Developed a dissemination plan that is feasible within your project constraints (e.g., budget, time)?**
yes

**Identified what team members have capacity to be responsible for executing your dissemination plan?**
yes

**Identified what resources are still needed and how you may get them to execute your dissemination plan?**
yes

# Plan For Sustainability

## What is the intended impact of sustaining your product?

| Selected options |
|---|
| Clinical and medical benefits (procedures & guidelines, tools & products) |
| Community and public health benefits (health promotion) |

## What do you need to sustain your product over time?

| Selected options |
|---|
| Money/funding stability |
| Partnerships |
| Organizational capacity (e.g., leadership, staff, systems) |
| Environmental support (e.g., champions, public support) |

| Products to prioritize |
|---|
| Environmental support (e.g., champions, public support) |
| Partnerships |

## Important Partners to prioritize for sustain the product

| Category | Type | Name |
|---|---|---|
| Patients and the public | Caregivers | Caregiver of a person with acquired brain injury |

## Your sustainability action plan

### Partnerships

| Question | Answer |
|---|---|
| Write a SMART objective that will help you to increase your capacity to address this need. | We will attend 4 community organization events to build relationships with patients and caregivers by Oct 2024 |
| Who will do the work? | Not answered |
| When will the work be done? | Not answered |
| What are the resources you still need to achieve this objective? | People |
| Use this space to detail assignments and tasks: | Not answered |

### Environmental support (e.g., champions, public support)

| Question | Answer |
|---|---|
| Write a SMART objective that will help you to increase your capacity to address this need. | We will conduct 4 meetings with organizational leaders to identify methods to incorporate the intervention into the organizational infrastructure by Jan 2025 |
| Who will do the work? | AR |
| When will the work be done? | By Jan 2025 |
| What are the resources you still need to achieve this objective? | People |
| Use this space to detail assignments and tasks: | Not answered |

## Evaluation Questions:

**Considered what needs you can feasibly address to have the greatest impact on sustaining your product?**
No answered yet.

**Collaboratively developed a plan that will build your team's capacity for sustaining your product?**
No answered yet.

# Evaluate Iteratively

## Evaluate your dissemination

### Podcast

**Target audience category: Patients and the public**
**Type of people to reach: Caregivers**

| Question | Answer |
|---|---|
| What is your evaluation question? | To what extent did the podcast increase awareness of the intervention for patients and their families? |
| What outcomes will you measure to answer your question? | Number of people reached, number of visits to the intervention website from the podcast link. |
| What types of data will you collect? | Web analytics |
| What resources do you need to measure your outcomes? Where will they come from? | Staff to evaluate analytics |
| When will you collect this information? | 3 consecutive months after podcast |

### Meeting (in-person/virtual)

**Target audience category: Practitioners**
**Type of people to reach: Mental health providers (psychiatrists, psychologists)**

| Question | Answer |
|---|---|
| What is your evaluation question? | Not answered yet. |
| What outcomes will you measure to answer your question? | Not answered yet. |
| What types of data will you collect? | Not answered yet. |
| What resources do you need to measure your outcomes? Where will they come from? | Not answered yet. |
| When will you collect this information? | Not answered yet. |

# Evaluate your Sustainability Plan

## Partnerships

### Your need:

People

### Your SMART Objective to increase capacity to address this need:

We will attend 4 community organization events to build relationships with patients and caregivers by Oct 2024

| Question | Answer |
|---|---|
| What outcomes would you expect from successfully completing this objective? | Increased interest in the intervention from individuals in these community organizations. |
| How will you get the information you need to know when you have been successful? | Number of individuals enrolled following attendance at the community events. |
| What resources do you need to measure this? Where will they come from? | Staff. |
| When will you collect this information? | Following each community organization event. |

# Environmental support (e.g., champions, public support)

## Your need:

People

## Your SMART Objective to increase capacity to address this need:

We will conduct 4 meetings with organizational leaders to identify methods to incorporate the intervention into the organizational infrastructure by Jan 2025

| Question | Answer |
| --- | --- |
| What outcomes would you expect from successfully completing this objective? | Not answered |
| How will you get the information you need to know when you have been successful? | Not answered |
| What resources do you need to measure this? Where will they come from? | Not answered |
| When will you collect this information? | Not answered |

# Evaluate your use of D4DS

| Question | Answer |
|---|---|
| Do you regularly communicate with and engage diverse partners in your work? | Not answered yet. |
| Has your work been responsive to partner priorities and needs? | Not answered yet. |
| Have you used your value proposition statement when communicating with key partners about the importance of this work? | Not answered yet. |
| Have you adapted your approach to better align with the settings, resources, workflows and broader social systems in which your work is done? | Not answered yet. |
| Did your dissemination products EQUITABLY reach all subgroups of your intended target audience within your goal timeline? | Not answered yet. |
| Has your target audience continue to use and benefit from your product over your goal time period (e.g., sustain use for 2 years)? | Not answered yet. |
| Have you developed an action plan to help you build capacity for sustaining your product? | Not answered yet. |
