# Supplementary material for: Supporting teams with designing for dissemination and sustainability: the design, development, and usability of a digital interactive platform
Source: Implement Sci. 2024 Dec 31;19:82. doi: 10.1186/s13012-024-01410-7 (PMC11686880; doi:10.1186/s13012-024-01410-7)
Supplement: Supplementary file 2 — Supplementary Material 2. [file 13012_2024_1410_MOESM2_ESM.docx]

| **Supplementary Material 2: List of Changes from Usability Testing** | |
| --- | --- |
| **Requested Change** | **Status** |
| **Home Page** | |
| Add a "view the education hub" button on the top bar of the landing page. | Completed |
| Change back button within the action planner to return you to the landing page | Completed |
| Change tagline under the D4DS planner to "The D4DS Planner will help you engage partners through a planning process to maximize the impact of your project.” | Completed |
| In parenthesis, add 3 examples (interventions, policy, evidence) for "the product" in the sentence "D4DS refers to a way of ensuring that “the product” of our work solves a problem and fits the context in which it is intended to be adopted." | Completed |
| The "tabs" at the top of page (e.g., "what is D4DS") are not being noticed. If we could add color (perhaps shades blue) to visualize those tabs. | Completed |
| When you click the "What is D4DS?" it doesn’t move anywhere. Could it navigate the user to the "introduction to D4DS video" section. | Completed |
| Re-size items (D4DS planner, What is D4DS box) to make sizes more equal/represent their importance within the tool | Completed |
| Add introductory video to the landing page and add text under the video that states "Watch this video for a short overview of D4DS" | Completed |
| Make it clear that users should scroll down | Completed |
| Make sure things that appear to be clickable are-- e.g., key definitions bounce when you hover, and the push pull figure. | Completed |
| Add introductory video to the landing page. | Completed |
| In figure for "What research products can the D4DS Planner help you disseminate and sustain?" change "technology and infrastructure" to just "technology" | Completed |
| Add text (see attached doc) to add what to expect and how to use the planer. | Completed |
| Reduce text (see attached doc) above Fit to Context Framework | Completed |
| **Login and Account Creation** | |
| Decrease time it takes to receive email to verify your account and add text for users to indicate that sometimes this takes a few minutes. | Completed |
| Make it clear if there are password requirements or not | Completed |
| Have name and email auto populate if user already has an account | Completed |
| **Project Set-up** | |
| Make project set-up button easier to find: increase font size, add blue plus sign at the top of the page and write out "add project" | Completed |
| Remove word limit on questions in project set up | Completed |
| Add other option for “What stage of the project you are in." Allow users to select multi answers. | Completed |
| Change "I will need guidance to get started using this planner" to "I would like to take a questionnaire to help me get started using this planner". | Completed |
| In the hover over in the star-wheel change to "Add or select a project to develop plans using the interactive features of the tool." In the my projects tab when a user has not created any projects yet, "This tool helps you develop a D4DS plan for a project you are already working on. You have not added any of your projects to the D4DS planner yet. Click the button below to add information about your project to allow you to use the interactive features of this tool." | Completed |
| Amend questions and eliminate multiple questions (see separate word document) to reduce user burden. | Completed |
| Add a slider bar with landmarks to allow users to enter their project budget | Completed |
| Add an information security statement once they create a project. | Completed |
| **Action Planner** | |
| Add description at the top of the on main change to orient users. | Completed |
| Add a guided tour of the tool for users when they first log-in. | Completed |
| Add video explaining the D4DS tool to the main page. "To get started, watch this video" | Completed |
| Most did not go to education hub or landing page independently when on Action Planner page. Use different colors for “Education Hub” and “Guidance” tabs to lead people to look up these sections of the tool. | Completed |
| Move "landing page" tab on left to top of list (below login and above action planner). Changing the name to "Home Page" | Completed |
| Add lines between the tabs and carrots on the menu a bit darker (hard to see) | Completed |
| **Guidance** | |
| Add icons next to action items so users relate these to the star-wheel | Completed |
| Add link to key definitions (on home page) | Completed |
| **Within Action Items** | |
| Move instructional videos to the start of each page. Above the video say "To learn about this action item" | Completed |
| In identify partners, the add button for potential partners is currently off to the right. Could you move button directly under potential partners to make more visible. | Completed |
| In identify partners, switch partner category and partner type questions to come first in the create new partner form. Add other option for category-- then add prompt for partner type "describe specific types of partners" | Completed |
| In Empathize and Outline the Problem, allow admins and collaborators to edit questions. - in case a question is edited, and that question has answers for various partners, DO NOT DELETE partner answers. | Completed |
| New activity for confirm and co-design your product to provide users with co-design methods. | Completed |
| Content changes (separate word doc) to the Dissemination planning action item that reduce words/complexity. | Completed |
| Where it says “Your responses are saved,” state specifically where the responses are saved on the tool so the user can go back and check if there’s need to edit or add more information. | Completed |
| Wording changes made throughout action items in separate documents. | Completed |
| **Education Hub** | |
| Only display titles with the full card being displayed using a carrot (drop down) | Completed |
| Create headings for each action items (with General at the top) and list resources alphabetically within each heading. For resources tagged with more than one action item, list them within each tagged action item. | Completed |
| Add a "content provided" tag of "Grant Section" | Completed |
| Add way to link a PDF resource or video located in the education hub in the dive deeper sections within action items. | Completed |
| Add key definitions from landing page here as well. | Completed |
